# The impact of value co-creation behavior on customers’ experiences with and loyalty to P2P accommodations

**DOI:** 10.3389/fpsyg.2022.988318

**Published:** 2022-10-05

**Authors:** Jing Lyu, Keyan Cao, Shan Yang

**Affiliations:** ^1^School of Management, Shenzhen Polytechnic, Shenzhen, Guangdong, China; ^2^School of Education, Shenzhen Polytechnic, Shenzhen, Guangdong, China

**Keywords:** P2P accommodations, customer experience, value co-creation, participation behavior, citizenship behavior

## Abstract

This study explores the impact of customers’ value co-creation behavior on their experiences with and loyalty to P2P accommodations. We propose a theoretical model integrating two lines of tourism research: customer value co-creation and customer experience. To extract the dimensions of customer experience and test the proposed model, 34 in-depth interviews were conducted along with a survey of Chinese Airbnb users. Structural equation modeling and mediation analysis were implemented to assess relationships involving customers’ value co-creation behavior, experience, and loyalty. Results indicate that customer citizenship behavior directly influences loyalty. In particular, relationships involving customers’ participation behavior and citizenship behavior with loyalty are both mediated by customer experience. Relevant implications and future research opportunities are discussed.

## Introduction

The customer experience represents a new form of post-service economic value, and many scholars have discussed its role in helping organizations gain a competitive advantage (Pine and Gilmore, 1998; Verhoef et al., 2009). In early studies of the customer experience, researchers generally defined *experience* from a utilitarian perspective with an emphasis on the functional value of experience (e.g., Abbott, 1955; Hauser and Urban, 1979). At that time, marketers maintained a goods-oriented mindset, positing that value is produced and embedded in goods through manufacturing processes and later destroyed by consumers. As goods and services become more commoditized, products and services that fulfill functional needs are no longer sufficient today; contemporary consumers’ demands go beyond the mere delivery and consumption of products and services as they seek “engaging, robust, compelling and memorable” experiences (Gilmore and Pine, 2002, p. 10).

Countering the goods-centered logic that has been well-established in management theory, Vargo and Lusch (2004) introduced the concept of service-dominant logic (SDL), which challenged the presumptions of mainstream marketing theory. According to SDL, customers are co-creators of value rather than targets of that value. Customers determine value in the consumption process, operating at the intersection of the service provider and customer (Lusch et al., 2007). SDL has risen to prominence as a new marketing perspective over the last decade to become a dominant narrative in collaborative marketing (Warnaby and Medway, 2015). The recent phenomenon of the collaborative economy exemplifies an exploration of SDL’s utility within a broader scope (Brodie et al., 2019). Hybridized production and consumption constitute the most salient feature of the collaborative economy (Cohen and Munoz, 2016).

Studies have shown that participation in collaborative consumption enables consumers to gain and maintain social relations emerging from sharing behavior (Belk, 2010). Interactions with hosts and local community members, coupled with active participation in co-creation activities, help elicit authentic, personalized, and memorable experiences (Mathisen, 2013). Sharing-business practitioners must therefore understand (a) which factors contribute to customers’ service evaluations and (b) how to engage customers in the value co-creation process. Only with this knowledge can service providers allocate resources effectively to achieve optimal return on investment.

Although the experiential nature of sharing accommodations has been widely studied (e.g., Guttentag, 2015; Tussyadiah and Zach, 2017), antecedents of the customer experience remain under-researched. Co-creation, which is widely considered conducive to memorable and unique customer experiences in tourism, is in the pre-theory stage and lacks empirical evidence (Chiadmi, 2014). Moreover, the customer’s role in co-creation has yet to be systematically examined in tourism research (Sugathan and Ranjan, 2019); scholars have called for additional studies examining how value is co-created (Busser and Shulga, 2018; Malshe and Friend, 2018). Value co-creation is viewed as an overarching component of collaborative consumption (Lusch et al., 2007), yet few studies have examined co-creation modalities in a sharing accommodation context. The question of which co-creation behaviors are most effective in creating higher-order customer experiences, and in turn fostering customer loyalty, must be answered.

This study attempts to fill these theoretical gaps by exploring the dynamics of customers’ value co-creation behavior and its impact on behavioral outcomes with a focus on the customer experience and loyalty in the context of sharing accommodation field. Specifically, we propose that customers’ value co-creation behavior determines customers’ perceptions of their experiences and thus influences their behavioral intentions. The research purposes of this study are (1) to examine the components which construct value co-creation behavior in the context of P2P accommodation, (2) to explore the experiential dimensions of customers’ perceptions of P2P accommodations, (3) to evaluate the influences of different value co-creation behaviors on customer experiences and loyalty, (4) to explore the mechanism of how value co-creation behavior exposes influences on customer loyalty, and (5) to provide insights and suggestions for sharing accommodation operators on how to improve guests’ experience and loyalty.

## Theoretical background

### Customers’ experiences with P2P accommodations

The P2P accommodation market is still in an early stage of development. Despite drastic growth within the past several years in terms of business and customer volume, little research has considered whether this shift has altered customers’ expectations and evaluations of accommodation services (Tussyadiah and Zach, 2017). As a new sharing-business mode, the services provided by P2P accommodations tend to be distinct from those offered at hotels. To better understand the factors differentiating P2P accommodations from long-established accommodation settings including hotels and B&Bs, scholars must explore the experiential dimensions that matter to customers when evaluating P2P accommodations.

Various studies (e.g., Tussyadiah and Zach, 2017; Guttentag et al., 2018; Lutz and Newlands, 2018; Tussyadiah and Pesonen, 2018; Zhu et al., 2019) have outlined dimensions of the customer experience in P2P accommodations, consisting of five major elements: the physical environment, service quality, guest-host relationship, peer guest interactions, and local cultural experience. These facets represent the primary dimensions of P2P accommodation experiences. Accordingly, customers’ evaluations of their P2P accommodation experiences (including their reflections upon relevant products and services) can be holistically captured in terms of cognition and affect.

The customer experience lies at the heart of the tourism industry, highlighted by a SDL that provides a conceptual framework delineating how the consumer has become pivotal to the development and marketing of tourism products through a process of co-creation with the producer (Shaw et al., 2011). Tourism operators have increasingly acknowledged the great extent to which visitors shape their own experiences (Mathisen, 2013). Moreover, an abundance of research has offered evidence of customers’ desire and demand to co-create experiences with service providers (e.g., Prahalad and Ramaswamy, 2004a; Im and Qu, 2017).

Many studies have explored motivational factors that drive customers to choose P2P accommodations (e.g., Guttentag, 2015; Tussyadiah and Pesonen, 2018), but few have examined the antecedents, consequences, and specific dimensions of the customer experience, particularly from a value co-creation perspective. As an emerging lodging market, P2P accommodations are quite different from the standard hotel market. Some studies have shown that B&B guests are particularly interested in uniqueness and novelty in their living experiences (Mcintosh and Siggs, 2005). Positive customer experiences and high customer satisfaction may not necessarily lead to customer loyalty. The interrelations among customer experience, perceived value, satisfaction, and loyalty must therefore be re-examined in the P2P accommodation market.

Furthermore, most research on the sharing economy has been conducted from a Western perspective and in Western regions (Cheng, 2016). More attention should be paid to emerging areas that present unique group dynamics. Airbnb is reportedly facing barriers to growth in China (Nextunicorn, 2019), but scholars have not empirically determined whether cultural resistance is responsible for this phenomenon. Therefore, to further develop the Chinese P2P accommodation market, researchers must evaluate how Chinese customers perceive such services; consumers’ experiences and satisfaction are essential to providing actionable implications for investors, operators, and other stakeholders.

### Customer value co-creation

#### The concept of value co-creation

The concept of value co-creation is closely related to the customer experience. Prahalad and Ramaswamy (2004b) asserted that true co-creation occurs when firms provide “experience spaces” where dialogue, transparency, and information access enable customers to engage in experiences that suit their needs and level of involvement. Customers create self-tailored experiences and value by integrating their own resources including expertise, skills, abilities, and capabilities with the provider’s network and customer network resources.

The distribution and exchange of commodities and manufactured products have dominated the marketing domain since its birth at the start of the 20th century (Lusch et al., 2007). Under this goods-dominant logic, which focused on tangible resources, services were considered a type of product or value-adding enhancement to tangible products; value was thought to be created by firms and distributed to consumers (Lusch et al., 2007). Over the past few decades, marketing has evolved toward a new dominant logic as perspectives have begun to focus on intangible resources, relationships, and value co-creation. Vargo and Lusch (2004) introduced the concept of SDL, wherein the customer is a co-creator of value; an enterprise cannot deliver value but only participate in creating and offering value propositions.

Researchers have defined value co-creation from a customer perspective (e.g., Prebensen et al., 2014; Tuan et al., 2019; Cui et al., 2022) and an organization/destination perspective (e.g., Prahalad and Ramaswamy, 2004b; Li and Petrick, 2008). Essentially, *value co-creation* represents the process by which customers create value for themselves through interacting with the experience environment by integrating organization-provided resources with their own cultural, emotional, and physiological resources. Co-creation is particularly relevant to the tourism industry. This field has been conceptualized as a type of performance embedded in social praxis (Perkins and Thorns, 2001). Rather than being passive sightseers, tourists now wish to “roll up their sleeves” (Eraqi, 2010, p. 79) and take active roles in their travel activities physically, emotionally, and intellectually. The performance turn in tourism argues that tourists are hungry to *do* rather than simply *see* (Eraqi, 2010; Assiouras et al., 2019). For example, from a tourist viewpoint, information seeking and idea generation constitute pre-consumption co-creation of the travel experience.

#### Customers’ co-creation behaviors in the sharing-economy context

Co-creation activities have become a top priority in tourism research (Shaw et al., 2011), especially in the context of the sharing economy. SDL is thought to explain the growing popularity of sharing-economy businesses (Heo, 2016). This concept emphasizes the importance of customer–service provider interaction (Vargo and Lusch, 2004; Bu et al., 2022). In the sharing economy, social interaction is one of the most important factors motivating tourists to use P2P accommodation rentals (Tussyadiah and Zach, 2017; Tussyadiah and Pesonen, 2018). Tourists enjoy the sharing economy because they can create value by interacting with hosts and the local community; that is, today’s consumers prefer to be active partners in value creation. Heo (2016) identified the need for a more extensive theoretical explanation of SDL and value co-creation relative to the new business phenomenon of sharing.

As SDL implies, customers are the core of value creation and have assumed new roles as collaborators in their service experiences. Some studies have explored dimensions of co-creation behaviors. For instance, Campos et al. (2018) reviewed the literature defining co-creation and the dimensions of co-creation behaviors in tourism and hospitality contexts. Specifically, they summarized two dimensions of on-site co-creation experiences: tourists’ active participation and interactions. Active participation refers to tourists’ engagement in an experience based on their personal resources, capabilities, and strategies during physical and cognitive activities (Morgan et al., 2009; Prebensen and Foss, 2011). Interactions refer to relationships between tourists and people that manifest during an experience (Lugosi and Walls, 2013).

Aside from on-site co-creation behaviors, other scholars have identified several factors in the co-creation experience that can occur before and after travel. For example, Camilleri and Neuhofer, 2017 analyzed Airbnb reviews in Malta and uncovered six themes related to value co-creation: arriving and being welcomed, expressing positive/negative feelings, evaluating the accommodation and location, interacting with and receiving help from hosts, recommending the accommodation to others, and thanking one another.

Based on early research, Yi and Gong (2013) outlined two types of customers’ value co-creation behavior, namely customer participation behavior and citizenship behavior. Customer participation behavior refers to (in-role) behavior necessary for successful value co-creation, and customer citizenship behavior is voluntary (extra-role) behavior that provides extraordinary value to the firm but is not necessarily required for value co-creation (Groth, 2005; Yi and Gong, 2013; Mitrega et al., 2022). This study also posits that customer participation behavior comprises four dimensions: information seeking, information sharing, responsible behavior, and personal interaction. In a similar vein, this study views customer citizenship behavior as consisting of feedback, advocacy, helping, and tolerance.

Researchers have largely examined the dimensions of customers’ value co-creation behavior from either a multidimensional perspective (e.g., Bettencourt, 1997; Bharwani and Jauhari, 2013; Bertella, 2014) or a uni-dimensional perspective using single-or multiple-item measures (e.g., Fang et al., 2008; Rihova et al., 2018). In an empirical study, Yi and Gong (2013) systematically explored the dimensionality of customers’ value co-creation behavior by developing a measurement scale. Their scale demonstrated internal consistency, construct validity, and nomological validity, indicating its suitability to measure value co-creation behavior in the service industry. We therefore assessed customers’ value co-creation behavior using this scale, although some measurement items were subject to revision in our study to reflect the hospitality literature and findings from in-depth interviews.

### Customer loyalty

Customer loyalty can be defined as a customer’s likelihood of returning to a hotel (Bowen and Shoemaker, 2003). It costs less for firms to serve loyal customers because these consumers are more familiar with a given product and service and thus require less information; additionally, long-term customers tend to buy more, bring in new customers, and be less price-sensitive than newer consumers (Reichheld and Sasser, 1990). Yang and Peterson (2004) noted that loyal customers are more willing to share positive word-of-mouth and spend extra money on specific service operations. As such, it is important to understand the aspects of business performance that transform customers into repeat purchasers (Wilkins et al., 2009).

A large body of literature has considered customer loyalty. Most research maintains a general consensus that repeat purchase behavior, even if derived from customer satisfaction, does not necessarily reflect genuine loyalty (Wilkins et al., 2009). Customers make repeat purchases for various reasons, such as convenience and lack of choice. In addition, customer loyalty is especially difficult to achieve in tourism because novelty seeking has been identified as a primary motivation for travelers engaging in tourism activities (Sugathan and Ranjan, 2019).

The construct of customer loyalty has been considered from two perspectives. Some researchers have defined loyalty in behavioral terms based on the purchase volume for a particular brand (Tranberg and Hansen, 1986), whereas others have framed loyalty as attitudinal (Jacoby and Kyner, 1973). Baloglu (2002) examined attitudinal and behavioral dimensions of customer loyalty, noting that attitudinal loyalty consisted of trust, psychological or emotional attachment, and switching costs while behavioral loyalty involved cooperation (e.g., willingness to help a company and work with it to achieve mutual goals) and word-of-mouth recommendations (e.g., promotion, positive feedback, and referrals).

## Conceptual framework and hypotheses development

Co-creation behavior has been found to exhibit significant and positive associations with customers’ perceived value and behavioral intentions in service settings (e.g., Shaw et al., 2011; Wang and Wan, 2012; Alqayed et al., 2022). Additionally, the influences of specific dimensions of value co-creation behavior on customers’ experiences, satisfaction, and loyalty have been widely examined. First, customer participation has been found to exert positive effects on customers’ perceptions of their overall experiences. For example, active participation is positively associated with service quality and memorable experiences (Cermak et al., 1994; Mathis et al., 2016; Campos et al., 2018). Cermak et al. (1994) empirically proved that participation is strongly tied to repurchase and referrals in some service settings. Chen et al. (2015) identified three components of customer participation in hospitality settings, indicating that these three components significantly influenced customer loyalty. Wang and Wan (2012) conducted an empirical study and pointed out that customers’ interactions with employees, products, and other customers positively affected experiential value and brand loyalty.

Second, according to Yi and Gong (2013), customer citizenship behavior includes four aspects: feedback, advocacy, helping, and tolerance. Customers who are engaged in citizenship behavior are believed to be active in providing feedback to service providers and recommending services to their friends and relatives. These consumers also tend to be willing to interact with and help other customers and are patient when adapting to different situations. Customers’ feedback to service providers regarding the physical environment and services, along with their tolerance and patience when encountering service failure, contributes to services that better suit patrons and elicit higher experiential satisfaction. Through advocacy and helping behavior, customers share positive information and advice with other customers while enjoying relational experiences with service providers and other consumers.

Based on prior literature, customers’ participation behavior and citizenship behavior should exert positive effects on the customer experience and loyalty. Hence, Hypotheses 1(a) and 1(b) and 2(a) and 2(b) are proposed:

*Hypothesis 1*: Customers’ value co-creation behavior [(a) participation behavior, (b) citizenship behavior] positively influences customer loyalty.

*Hypothesis 2*: Customers’ value co-creation behavior [(a) participation behavior, (b) citizenship behavior] positively influences the customer experience.

Many studies have supported positive and significant relationships among the customer experience and its dimensions relative to customer satisfaction and loyalty (e.g., Zins, 2002; Han and Ryu, 2009; Wilkins et al., 2009; Wu and Liang, 2009). For example, Zins (2002) conducted an empirical study involving face-to-face interviews with leisure travelers and examined the relationships among customers’ consumption emotions, service experience evaluations, and satisfaction. Findings revealed that service experience evaluations positively influenced customer satisfaction.

Ample studies have also focused on partial dimensions of experience and its effects on customer satisfaction and customer loyalty. Han and Ryu (2009) examined relationships among three components of the physical environment (décor and artifacts, spatial layout, and ambient conditions), price perceptions, customer satisfaction, and customer loyalty in the restaurant industry. They found that these three physical environmental factors strongly influenced customers’ price perceptions and thus enhanced customer satisfaction and directly/indirectly influenced customer loyalty. Wu and Liang (2009) argued that the interactive relationship between customers and service employees is important in consumer evaluations. Furthermore, service employees’ behavior was identified as a key determinant of perceived service quality and consumer satisfaction. We therefore assume that the dimensions of the customer experience should collectively and individually influence consumers’ extent of satisfaction and loyalty. Studies have shown that in the hospitality industry, the relationship of “customers’ value co-creation behavior → customer experience → customer loyalty” is presumably positively related, leading to the following hypotheses:

*Hypothesis 3*: Customers’ experiences with P2P accommodations positively influence customer loyalty.

*Hypothesis 4*: Customers’ experiences mediate the relationship between customers’ value co-creation behavior [(a) participation behavior, (b) citizenship behavior] and customer loyalty.

**Figure 1:**
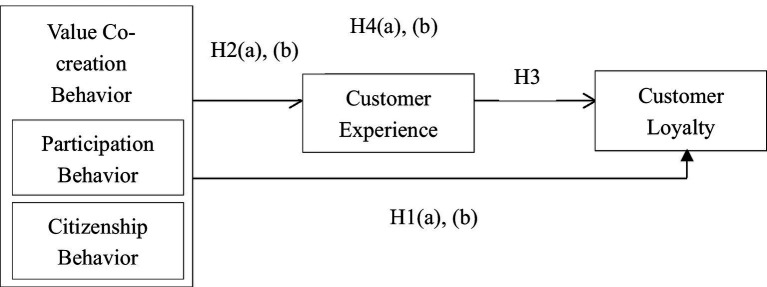
Hypothesized mediation model. **(A)** Participation behavior. **(B)** Citizenship behavior.

Based on the dimensions of the P2P accommodation experience drawn from the literature, and customers’ perceptions obtained from interviews, a measurement scale pertaining to customers’ experiences with P2P accommodations was developed following Churchill Jr (1979) scale development approach. Apart from examining the dimensionality of the customer experience, we tested interrelationships among the three focal constructs. We adopted valid and reliable measurement scales to assess customer co-creation behavior and loyalty. Figure 1 depicts relevant interrelationships drawn from the literature. Causality among constructs is indicated by arrows, which also show the direction of influence. The model begins with *customers’ value co-creation behavior*, evaluated using Yi and Gong (2013) measurement scale. The *customer experience* is predicted by the clues (i.e., major dimensions) of experience specified earlier. Customers’ value co-creation behavior influences the customer experience and resultant *customer loyalty*. As a consequent construct, customer loyalty is influenced by all other constructs. We developed this model primarily based on theory (i.e., the model components were derived from prior research and were chosen to address our study objectives).

## Methodology

### Research setting and data collection

We adopted a combination of qualitative and quantitative methods in this study. Data were collected through three research steps: in-depth interviews, a pilot study, and the main survey. Initially, 34 in-depth interviews were conducted using convenience sampling from March to May 2020; interviews were intended to elicit dimensions and aspects of customers’ experiences with P2P accommodations. A semi-structured interview format was used to explore attributes that can contribute to positive/negative experiences with P2P accommodations. All attributes derived from interviews were integrated with factors summarized from the literature, which served as the foundation for questionnaire development. All interviewees and survey respondents met two criteria: (1) Chinese adult travelers residing in China who (2) had used P2P accommodations within the past 6 months at the time this survey was conducted. The interview result is presented in Table 1.

**Table 1:** Interview results of customers’ experiences with P2P accommodations–dimensions and items (*N* = 34).

Dimensions	Sample quotes
**1.Physical environment**	
Cleanliness and tidiness	Clean, tidy, hygienic
Room size	Spacious room, suite room, entire house; nice for family stay
Facilities	Well-furnished, kitchen, washing machine, speedy WIFI, recreational facilities
Washing supplies	“The washing supplies were good-quality branded products”
Safety	“The room key used a password, which made me feel safe”
Interior design and decoration	Stylish and unique; exquisite design
Reliability	“The room design was the same as shown online”; “The room was not as spacious as shown online”
**2.Location**	
Convenience	Located in a central area; close to train station/attractions; convenient transportation and access
Surrounding environment	Natural and quiet surroundings
Nearby facilities	Supermarket or local market nearby; local food restaurants
**3.Sensory perceptions**	
Homelike feelings	Feel at home; warm, homelike feelings
Atmosphere with literature and art	Atmosphere with literature and art; full of artistic ambiance
Lighting	“Soft lighting makes me feel cozy and relaxed”
Smell	“The room is equipped with an aroma diffuser, and it was turned on before we arrived”
**4.Service quality**	
Daily room cleaning	“There was no service staff to clean the room for us, and we needed to take the garbage out ourselves”
Pick-up service	Pick-up service is provided
Friendly service staff	“The service staff were very friendly and polite; they smiled and greeted us whenever encountered”
Responsive	Service staff are conscientious and responsive
**5.Guest–host relations**	
Reliable	“Our flight was delayed, and the host waited for us until midnight”; “We booked the room online successfully, but the host said no room was available when we arrived”
Approachable	“The host didn’t appear”; “The host lived upstairs and greeted us every day”
Welcoming	Warmly welcomed by the host; “The host prepared lemon pie for us as a welcome dessert”
Eager to help	The host gives travel advice and recommends restaurants; the host helps us book tickets
Interaction	“We chatted with the host and shared personal stories”; “The host took us to the local market”
Care	The host cares about the guests; the host takes care of guests like family
**6.Interaction with peer guests**	
Communication	Review other guests’ comments; ask for other guests’ advice
Activities	Enjoy a barbecue; cook meals together; outdoor activities
Sharing	Chat and share travel experiences; share personal stories and make friends
**7.Local cultural experiences**	
Local food	The host cooks local food for the guests
Local people	Live with local people; the host speaks the local dialect
Room design	The room contains local cultural elements

A pilot study was performed next to collect quantitative data and purify the measures. Three hundred questionnaires were distributed to respondents based on convenience sampling, and 254 valid questionnaires were returned. Measurement items corresponding to constructs in the questionnaire were purified based on the results of the pilot study. The main survey was conducted during June and July 2020. Given our large sampling requirements, we collaborated with a reputable survey company^1^ to distribute the revised questionnaires to 800 P2P users. We adopted a quota sampling method to obtain a representative sample; respondents residing in first-tier cities in China (Beijing, Shanghai, Guangzhou, and Shenzhen) were selected as the target population. Ultimately, 519 complete questionnaires were returned.

### Construct measures

Measurement items related to the customer experience were derived from our interviews and previous studies. The initial measure for this construct included 37 items covering seven dimensions: physical environment, location, sensorial perceptions, service quality, guest–host relations, interaction with peer guests, and local cultural experiences. In addition to exploring the dimensions of customers’ experiences with P2P accommodations, in-depth interviews investigated dimensions of customers’ value co-creation behavior before, while, and after staying in P2P accommodations. Interviews helped verify Yi and Gong (2013) proposed dimensions and measurement items related to such behavior. Measurement items for customers’ value co-creation behavior are presented in Appendix I. Items for the customer loyalty construct were adapted from scales in relevant literature (see Appendix II).

### Data analysis

Scale development related to customers’ experiences with P2P accommodations was conducted in line with Churchill Jr (1979) recommendations. First, text data gathered from interviews were analyzed using grounded theory as suggested by Strauss and Corbin (1997). Open coding and axial coding were then performed to identify underlying uniformities in the original category set and to formulate a smaller set of higher-level concepts. Items extracted from interviews were added to the item pool derived from the literature. Member checking and a panel review were conducted to enhance validity. Second, based on data from our pilot study, exploratory factor analysis (EFA) was applied to explore the dimensionality of each construct under investigation. The measurement items were further revised according to the EFA running result to suit the study context. Third, confirmatory factor analysis (CFA) was executed using IBM SPSS AMOS 21 software with data collected from the main survey. We performed CFA to confirm the factors extracted from EFA using data from the pilot study and to verify latent constructs based on the observed variables (Byrne, 2010). The structural parameters in the empirical model were ultimately tested *via* structural equation modeling (SEM).

## Results

### Demographic profile

Main survey respondents’ demographic profiles were fairly diverse in terms of gender, age, marital status, occupation, education, and annual income. In terms of gender, 48% of respondents were men and 52% were women. Most were millennials: 73% were between 23 and 37 years old. Slightly more than half (57%) were single. Respondents’ occupations were well balanced, with every occupation option appearing in our sample. The respondents were mostly well educated: 82.6% had received more than a high school diploma. Respondents’ annual income generally fell between 30,000 and 120,000 RMB.

Regarding travel-and accommodation-related information, most respondents reported traveling for tourism purposes, either individually or with friends. Only 12.3% stated having traveled for business purposes. Most respondents (62%) had stayed at an Airbnb property for only one or two nights; 81.7% stayed at an Airbnb property costing less than 500 RMB per night.

### Measurement models

After purification through EFA, 5 dimensions were identified as comprising 30 measurement items related to the customer experience: *Tangible and sensorial experience* (Tang), *Host* (Host), *Cultural experience* (Cult), *Interactions with peer guests* (Inte), and *Location* (Loca). Our measurement model was tested using first-order CFA, and the results reflected a good model fit (χ^2^/*df* = 1623.96/395 = 4.111 < 5; TLI = 0.891; IFI = 0.902; CFI = 0.901; RMSEA = 0.078 < 0.08).

EFA results revealed two dimensions of value co-creation behavior, which consisted of five sub-dimensions: *Responsible behavior* (Resp), *Information sharing* (Info), *Advocacy* (Advo), *Feedback* (Feed), and *Tolerance* (Tole). CFA results reflected a good fit between the five-factor model and our data (χ^2^/*df* = 1158.38/289 = 4.008 < 5; TLI = 0.920; IFI = 0.929; CFI = 0.929; RMSEA = 0.076 < 0.08).

The reliability and validity of the measurement scales for customer experience and value co-creation behavior were examined next. The composite reliability of our multi-item scales was measured using Cronbach’s alpha. In this study, alpha coefficients ranged from 0.792 to 0.972 (customer experience) and from 0.861 to 0.973 (value co-creation behavior), well above the cut-off of 0.7 to suggest scale reliability (Hair et al., 2010). Validity is used to measure the adequacy of a measurement scale in measuring a specific variable (DeVellis, 2003); convergent validity and discriminant validity are most common. The correlation coefficient and average variance extracted (AVE) value for each dimension of customer experience and value co-creation behavior appear in Tables 2, 3, respectively. All dimensions were significantly related, with the correlation coefficient ranging from 0.621 to 0.801 (for customer experience) and from 0.651 to 0.860 (for value co-creation behavior). Therefore, customer experience and value co-creation behavior each demonstrated good convergent validity (Hair et al., 2010). The AVE value of each dimension exceeded 0.7, indicating good convergent validity (Bagozzi and Yi, 1988). In addition, the AVE value of each construct was greater than the squared correlation coefficient between corresponding constructs, confirming discriminant validity (Fornell and Larcker, 1981).

**Table 2:** Correlation matrix of five dimensions of customer experience.

	Customer experience	Tang	Host	Cult.	Inte.	Loca.
Customer experience	1					
Tang	0.949^**^	1				
Host	0.923^**^	0.801^**^	1			
Cult.	0.872^**^	0.758^**^	0.787^**^	1		
Inte.	0.823^**^	0.710^**^	0.736^**^	0.760^**^	1	
Loca.	0.833^**^	0.786^**^	0.716^**^	0.660^**^	0.621^**^	1
AVE		0.794	0.722	0.850	0.756	0.853

** *p* < 0.01.

**Table 3:** Correlation matrix of five sub-dimensions of value co-creation behavior.

	Value co-creation behavior	Resp.	Advo.	Info.	Feed	Tole
Value co-creation behavior	1					
Resp.	0.928**	1				
Advo.	0.924**	0.777**	1			
Info.	0.908**	0.834**	0.768**	1		
Feed	0.910**	0.785**	0.860**	0.772**	1	
Tole.	0.796**	0.651**	0.758**	0.621**	0.713**	1
AVE		0.848	0.762	0.721	0.768	0.795

** *p* < 0.01.

### Testing for reliability, validity, and structural model

We employed SEM to examine whether our five hypotheses were empirically supported. The structural model was constructed to determine the respective impacts of two dimensions of value co-creation behavior. In this model, customers’ participation behavior and citizenship behavior were taken as independent variables. Customer experience was considered a mediating variable between value co-creation behavior and customer loyalty. Table 4 lists path coefficients in the structural model. Our results reflected a good model fit (χ^2^/*df* = 4.193 < 5; TLI = 0.968; GFI = 0.946; CFI = 0.978; RMSEA = 0.079 < 0.08).

**Table 4:** Path coefficients in structural model.

Structural model (*N* = 519)			Standardized estimate	SE	*t*-value	*p*
Experience	**←**	Citizenship	0.274	0.39	2.823	**
Experience	**←**	Participation	0.561	0.187	5.683	***
Tangible	**←**	Experience	0.9			
Host	**←**	Experience	0.9	0.022	31.504	***
Cultural	**←**	Experience	0.866	0.012	28.806	***
Interaction	**←**	Experience	0.812	0.01	25.193	***
Location	**←**	Experience	0.803	0.01	24.685	***
Loyalty	**←**	Experience	0.262	0.017	4.819	***
Tolerance	**←**	Citizenship	0.792			
Feedback	**←**	Citizenship	0.915	0.059	24.714	***
Advocacy	**←**	Citizenship	0.941	0.086	25.675	***
Information	**←**	Participation	0.908			
Responsible	**←**	Participation	0.918	0.04	32.683	***
Loyalty	**←**	Citizenship	0.473	0.117	5.193	***
Loyalty	**←**	Participation	0.107	0.059	1.093	0.274

*** *p* < 0.001;

** *p* < 0.01.

Composite reliability of the multi-item scales was measured using Cronbach’s alpha; the alpha value for each construct appears in Table 5. All alpha coefficients were above the threshold of 0.7, indicating acceptable reliability. Convergent validity was substantiated by all AVE values exceeding 0.5 (see Table 4). Additionally, the AVE for each construct was greater than the squared correlation coefficients between corresponding constructs, verifying discriminant validity.

**Table 5:** Correlation (squared correlations), reliability, AVE, and mean values.

	Customer experience	Participation	Citizenship	Loyalty
Customer experience	1			
Participation	0.747 (0.56)	1		
Citizenship	0.73 (0.53)	0.783 (0.61)	1	
Loyalty	0.702 (0.49)	0.714 (0.51)	0.751 (0.56)	1
Cronbach’s α	0.972	0.958	0.951	0.898
AVE	0.68	0.68	0.65	0.67
Mean	4.85	5.16	4.98	5
Std. Dev.	1.34	1.35	1.32	1.32

All correlations significant at *p* < 0.01.

### Hypothesis testing

**Figure 2:**
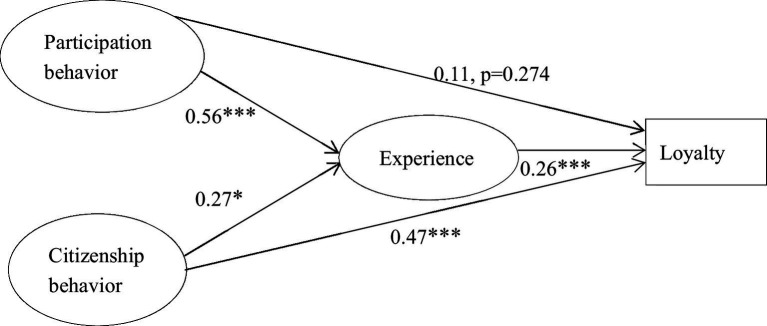
Structural model of customers’ experiences with P2P accommodations.

SEM results for this model are shown in Figure 2. Customer participation behavior was found to exert a significant positive influence on the customer experience (β = 0.56, *p* < 0.001). Customer participation behavior had a positive but non-significant influence on customer loyalty (β = 0.11, *p* = 0.274). Customer citizenship behavior had a significant positive influence on the customer experience (β = 0.27, *p* < 0.05) and customer loyalty (β = 0.47, *p* < 0.001). The customer experience had a positive and significant influence on customer loyalty (β = 0.26, *p* < 0.001).

In examining the indirect effects of value co-creation behavior (Hypothesis 4) (i.e., customers’ participation behavior and citizenship behavior) on loyalty *via* customer experience, we used the bootstrapping method with a 95% confidence interval (CI) and 10,000 resamples (Shrout and Bolger, 2002). In this case, indirect effects are significant when the 95% CI does not include zero. This bootstrapping method is considered superior to the Sobel test given its robustness in testing mediation effects (Montoya and Hayes, 2017). To assess indirect effects *via* bootstrapping, we interpreted the PROCESS macro (Model 4) (Hayes, 2013) for each model. The direct effect of participation behavior on loyalty was not significant, whereas the direct effect of citizenship behavior on loyalty was significant. Having established direct effects [Hypotheses 1(a) and 1(b)], indirect effects were then verified; see results in Table 6 The indirect effects of participation behavior on loyalty [β = 0.284, SE_boot_ = 0.065, 95% CI: (0.170, 0.422)], and citizenship behavior on loyalty [β = 0.240, SE_boot_ = 0.055, 95% CI: (0.143, 0.354)] *via* customer experience were all significant, providing support for Hypotheses 4(a) and 4(b), respectively. These findings indicate that the customer experience has a direct positive effect on loyalty and mediates the relationship between value co-creation behavior and loyalty.

**Table 6:** Empirical results of proposed hypotheses.

Hypothesized path		β			Results
H1a	Participation behavior → Customer loyalty	0.065, *t* = 0.274			Rejected
H1b	Citizenship behavior → Customer loyalty	0.473***, *t* = 5.193			Supported
H2a	Participation behavior → Customer experience	0.561***, *t* = 5.683			Supported
H2b	Citizenship behavior → Customer experience	0.274*, *t* = 2.823			Supported
H3	Customer experience → Customer loyalty	0.262***, *t* = 4.819			Supported
	**Mediating effects**	**Β**	**SE**_**boot**_	**95% CI**	Supported
H4a:		Participation behavior → Experience → Loyalty	0.284	0.065	0.0170, 0.422	Supported
H4b:		Citizenship behavior → Experience → Loyalty	0.240	0.055	0.143, 0.354	Supported

CI, confidence interval; boot, bootstrap.

* *p* < 0.05;

** *p* < 0.01;

*** *p* < 0.001.

## Conclusion

### Theoretical implications

This study makes several contributions to the tourism and hospitality management literature. First, our work extends the research stream on customers’ experiences in a P2P accommodation context by exploring the experiential dimensions of customers’ perceptions of P2P accommodations. As noted in previous research, the tangible and sensorial experiences, host, and location of P2P accommodations have been widely considered the most motivating attributes for visits. Our study also revealed two additional experiential dimensions, namely cultural experience and interaction with peer guests, which echo several other studies (e.g., Tussyadiah and Pesonen, 2016; Lutz and Newlands, 2018; Lin et al., 2019). Furthermore, apart from simply proposing experiential dimensions of P2P accommodations, our study takes a further step by developing a measurement scale of the customer experience and proposing the weight of each dimension in determining customers’ experiential evaluations.

Second, this study extends the body of knowledge around customer behavior in a P2P accommodation context by focusing on consumers’ value co-creation behavior and its impact on their experiential evaluations and behavioral intentions. Previous research tended to focus on identifying the factors that drove tourists to use P2P accommodations. As a response to Busser and Shulga’s (2018) call for research, we sought to better understand how value is created and what roles customers play in the value creation process. Our findings offer empirical evidence for the propositions that customers’ participation behavior and citizenship behavior have positive effects on producing memorable experiences. These aspects of consumers’ roles have thus been highlighted as antecedent variables of the customer experience.

Third, our study applied the SDL paradigm and incorporated the concept of value co-creation into P2P accommodation studies. The main contribution of our work lies in modeling the components and dynamics of value co-creation behavior and the customer experience in the context of sharing accommodations and exploring how these two constructs contribute to the ultimate marketing goal of customer loyalty. Our study is the first to consider the full dynamics of value co-creation behavior within a single model. As the P2P accommodation industry is still in its infancy, our findings are significant in opening avenues for future research by proposing a new pathway from value co-creation behavior to customer loyalty.

### Practical implications

Our findings offer practical implications for P2P accommodation stakeholders and their competitors. By identifying the different influences of customers’ value co-creation behaviors (customer citizenship behavior and participation behavior), this study could assist peer managers to comprehend the role of peers/guests as value co-creators, and the main source of benefit to P2P properties and platforms.

First, this research provides a roadmap for sharing accommodation operators to design and manage customers’ experiences. The important role of value co-creation has been highlighted in this study as essential in creating satisfying experiences and promoting consumer loyalty. As customers gain power and control, P2P accommodation platforms, and service providers should focus on building an experiential environment conducive to provider–customer dialogue in order to increase co-creation experiential value. Communication and interaction among guests, service providers, and local communities, whether occurring face-to-face or virtually, also play important roles in customers’ value co-creation behavior. On one hand, P2P accommodation platforms should apply new technologies such as augmented reality, interactive maps, and smart communications (Buonincontri et al., 2017) or establish reputable rewards for knowledge sharing (Chen et al., 2017) to facilitate direct interaction and information sharing among guests and hosts. On the other hand, to encourage customers’ active participation, hosts should consider designing and facilitating offline activities to offer guests immersive destination experiences and interactive opportunities, such as cooking courses or traditional craft workshops. Guests can exert indirect or direct impacts on the co-creation of other guests’ experiences (Tung et al., 2017; Assiouras et al., 2019).

Second, customers’ willingness to co-create varies by age, cultural background, and consumption behavior (Buonincontri et al., 2017). As Chathoth et al., 2013 discovered, some tourist groups may be unwilling to participate in co-creation activities because they do not recognize the value of active participation in experience creation. Similarly, in a study by Lyu et al. (2019), Airbnb guests older than age 40 were found to express less interest in socializing with other guests. Some customers may also be reluctant to co-create due to a lack of knowledge and self-efficacy.

Given these findings, service providers should segment their markets based on customers’ willingness and competence and then provide guests with different combinations of active participation, interaction, and sharing possibilities (Buonincontri et al., 2017; Im and Qu, 2017). Meanwhile, service providers should be reminded that the co-creation process must be managed appropriately to avoid “overburdening” customers (Stokburger-Sauer et al., 2016, p.566) or contributing to “value co-destruction” (Plé and Cáceres, 2010, p.431); otherwise, co-creation activities may detract from customers’ overall experience. This pattern may explain the false direct correlation between participation behavior and loyalty identified in this study.

Third, tourist pursue pleasure as well as core meaning in travel, which includes escaping from daily life, personal development, and re-establishing interpersonal relationships (Pine and Gilmore, 1998). Social and cultural experiences are contingent on guests’ subjective purposes. Customers’ value co-creation behavior was found to positively influence the customer experience. To use P2P accommodations to the fullest, guests are encouraged to seek related information online and to communicate with hosts before making reservations. Active participation in an experience and interaction with others contribute significantly to enhanced attention and memorability of a tourist experience (Campos et al., 2018). Guests can actively participate in value co-creation activities through various means, such as by searching for information about a service, sharing feedback with service providers, assisting other customers if they need help, and so on. P2P accommodation platforms and hosts should develop a friendly environment (online and offline) to facilitate guests’ engagement in creating an experience and associated value.

## Limitations and directions for further research

Similar to other studies, this research is not free of limitations. Our results should be interpreted cautiously for several reasons. This study is the first to apply customers’ value co-creation behavior as an antecedent of experience and loyalty in the P2P accommodation industry. Because the customer experience is highly subjective and may differ across cultures, the findings from our model may not be generalizable to other settings. Future research should replicate this model in other contexts to cross-validate our results. Data for the proposed model were also cross-sectional and correlational; all predictor and outcome variables were obtained from the same population, and our interpretations are therefore tentative. Future research could address these limitations by using longitudinal analysis to capture and control disparities and causal directions among variables. In addition, measurement items for the construct of value co-creation behavior were derived from prior hospitality and marketing studies; other potential items may be discovered when adopting other methods, such as qualitative research or big data analysis. To expand upon the model proposed in this study, subsequent research should include other variables such as the perceived value of experience (Knutson et al., 2010; Wu, 2013), motivational factors behind staying in P2P accommodations (Guttentag et al., 2018), and customer satisfaction (Huang and Hsu, 2010) to explore the relationship of value co-creation behavior and loyalty. As disclosed in a recent market research report from China Tourism Academy (2017), when selecting Airbnb properties, female tourists emphasize their cultural and emotional experiences while male tourists focus more on practical aspects (e.g., the safety and convenience of the property). As such, future research could deepen our proposed model by incorporating certain sociodemographic variables (e.g., gender) as moderators and testing whether they moderate the relationship between value co-creation behavior and loyalty.

## Data availability statement

The original contributions presented in the study are included in the article/supplementary material, further inquiries can be directed to the corresponding author.

## Author contributions

JL was in charge of conceptualization, writing-original draft, and funding acquisition. KC was in charge of investigation. SY was in charge of formal analysis, writing-review and editing, and funding acquisition. All authors contributed to the article and approved the submitted version.

## Funding

This work was supported by Research Startup Foundation for Advanced Talents provided by Shenzhen Polytechnic (Project No. 6021310017S) and the Innovation Team Project of Universities in Guangdong Province, China (grant no. 2021WCXTD026).

## Conflict of interest

The authors declare that the research was conducted in the absence of any commercial or financial relationships that could be construed as a potential conflict of interest.

## Publisher’s note

All claims expressed in this article are solely those of the authors and do not necessarily represent those of their affiliated organizations, or those of the publisher, the editors and the reviewers. Any product that may be evaluated in this article, or claim that may be made by its manufacturer, is not guaranteed or endorsed by the publisher.

## Footnotes

^1^ www.sojump.com

## Appendix

APPENDIX I Measurement items of customers’ value co-creation behavior used in the survey.

**Table:**

*Information seeking*
I have asked others for information on what this service offers
I have searched for information on where this service is located
I have paid attention to how others behave to use this service well
*Information sharing*
I clearly explained what I wanted the host to do
I gave the host proper information
I provided necessary information so the host could perform his or her duties
I answered all employees’ service-related questions
*Responsible behavior*
I performed all tasks that were required
I adequately completed all expected behaviors
I fulfilled responsibilities to the business
I followed the host’s directives or orders
*Personal interaction*
I was friendly to the host and service staff
I was kind to the host and service staff
I was polite to the host and service staff
I was courteous to the host and service staff
I was not rude to employees
*Feedback*
If I had a useful idea on how to improve service, I let the host know
When I received good service from the host, I spoke up about it
When I experienced a problem, I let the host know about it
*Advocacy*
I said positive things about XYZ and the host to others
I recommended XYZ and the host to others
I encouraged friends and relatives to use XYZ
*Helping*
I assisted other customers if they needed my help
I helped other customers if they seemed to have problems
I taught other customers to use the service correctly
I gave advice to other customers
*Tolerance*
If service was not delivered as expected, I was willing to put up with it
If the employee made a mistake during service delivery, I was willing to be patient
If I had to wait longer than I normally expected to receive the service, I was willing to adapt

APPENDIX II Measurement items of customer loyalty used in the survey.

**Table:**

Construct measurement items	
Customer loyalty	I would recommend this accommodation to my friends and relatives
	I would say positive things about this accommodation
	I would return to this accommodation if I were back in this area
